# Persistent cough in a 61-year-old male

**DOI:** 10.4103/1817-1737.38050

**Published:** 2008

**Authors:** Kevin O'Regan, Sean McSweeney, Jamal Al Deen Alkoteesh

**Affiliations:** *Department of Diagnostic Imaging, Cork University Hospital, Wilton, Cork, Ireland*

## Case History

A 61-year-old male, a former tunneler, presented with progressive shortness of breath and persistent productive cough.

### Examination findings

Auscultation revealed coarse crepitations in the mid-zones bilaterally with mild generalized wheeze. Examination was otherwise unremarkable. Pulmonary function testing showed decreased lung compliance, reduced FEV_1_ and FEV_1_/FVC ratio and reduced TL_CO_= Diffusion capacity for carbon monoxide.

## Questions

**Figure 1:**
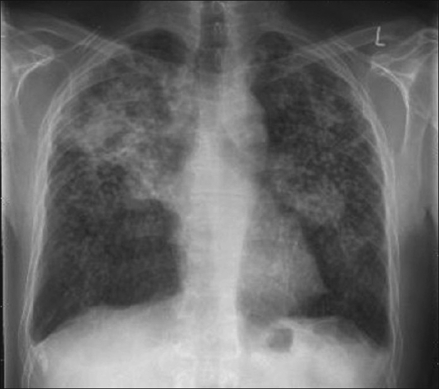
PA CXR

**Figure 2:**
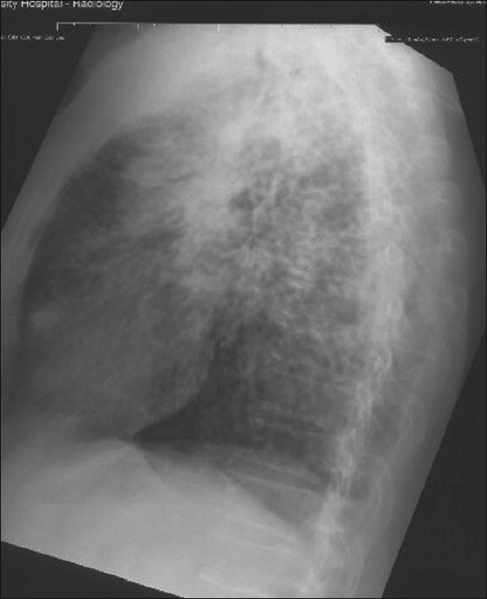
Lateral CXR

**Figure 3:**
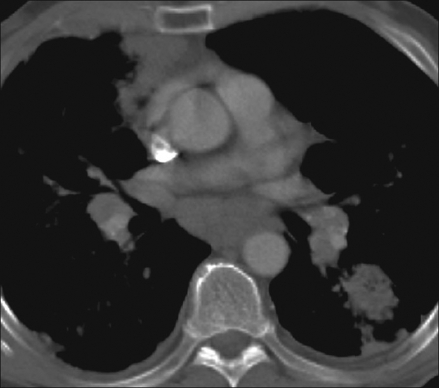
Contrast-enhanced CT (Mediastinal window settings)

**Figure 4:**
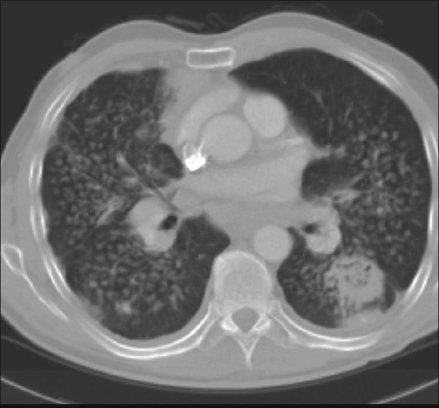
Contrast-enhanced CT (Lung window settings)

1. What are the findings on the chest radiograph and CT of thorax [Figures 1-5]?

**Figure 5:**
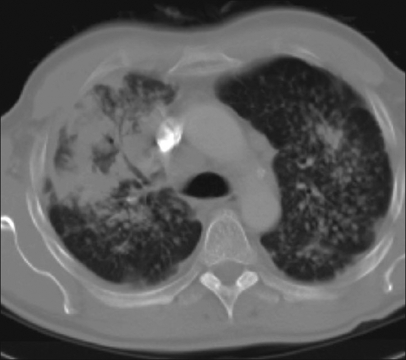
Contrast-enhanced CT (Lung window settings)
2. What is the differential diagnosis?
3. What further investigations would you perform to confirm the diagnosis?

## Answers

1. The chest radiograph [Figures 1 and 2] shows bilateral, sub-centimeter, nodular opacities predominantly located in the mid and upper zones. There are confluent larger opacities in the right upper zone and in the left hilar region. Mild hyperinflation of both lower lobes is also seen. The chest CT [Figures 3-5] shows mediastinal and bilateral hilar lymphadenopathy with peripheral (‘eggshell’) calcification in a number of lymph nodes (arrow). Multiple nodules are seen in both upper lobes with large, confluent masses containing air bronchograms. Cavitation is also seen in the soft tissue mass in the right upper lobe.
2. The differential diagnosis includes silicosis, tuberculosis, pulmonary malignancy, sarcoidosis, rheumatoid nodules (Caplan's syndrome) and pulmonary alveolar proteinosis.
3. A significant history of occupational exposure to silica, along with characteristic radiological findings and absence of other likely conditions, is sufficient to make a diagnosis of silicosis. Other findings in the history, such as constitutional symptoms, fever, weight loss, etc., should prompt further investigations to evaluate for TB, sarcoidosis or malignancy. In difficult cases, bronchoalveolar lavage, transbronchial or open-lung biopsy may be required to make the diagnosis.

## Diagnosis

Progressive massive fibrosis secondary to chronic silicosis.

## Discussion

Pulmonary silicosis results from the inhalation of crystalline silica. Exposure to silica is associated with a number of occupations, including underground coal mining, hard rock mining, tunneling, quarrying, sandblasting and glass manufacturing.[1] Toxicity results from production of inflammatory cytokines secondary to epithelial cell injury, leading to inflammation and ultimately, fibrosis.[1]

Two main clinical forms of silicosis exist: acute and chronic. Acute silicosis is seen following exposure to high silica concentrations, and symptom onset is within a few weeks to a few years. Symptoms include cough, weight loss, fatigue and chest pain. The radiographic features of acute silicosis are quite distinct from the chronic form, consisting of bilateral, mainly perihilar, consolidation and ground-glass opacification.[2] Patchy and nodular ground-glass opacities are seen on high resolution computed tomography with consolidation and occasionally a ‘crazy paving’ appearance.[2] The prognosis in acute silicosis is poor, with rapid development of respiratory failure and cor pulmonale.

The chronic form, which is more common, of the disease develops 10 to 30 years following the first exposure to silica. The radiographic hallmark is of multiple silicotic nodules which are composed of a central zone of collagen, surrounded by a rim of particle-laden macrophages. These are easily identified on chest radiograph and on CT and tend to be predominantly located in the upper lobes and in the posterior segments of the lung.[3] Calcification of these nodules occurs in 10-20% of patients.[2]

Other findings include hilar and mediastinal lymph node enlargement, often with a characteristic peripheral ‘eggshell’ calcification (seen in 56%) and pleural thickening.[3]

A minority of patients develop a more severe and accelerated form of chronic silicosis, known as complicated silicosis or progressive massive fibrosis. This is associated with exposure to high levels of silica; and the clinical picture is variable, ranging from asymptomatic to severe respiratory compromise.

Progressive massive fibrosis develops from coalescence of silicotic nodules to form large, asymmetrical, upper-lobe opacities greater than 1 cm in diameter. These opacities have an irregular outline and often contain calcification and air bronchograms. Cavitation may develop in these lesions, although its presence is usually indicative of concomitant mycobacterial infection.[2]

There is an increased incidence of lung cancer by 30% or more[4] in patients with silicosis, and silica has been listed as a carcinogen. Patients with silicosis also have increased susceptibility to mycobacterial infection, with tuberculosis occurring in 25% of patients with acute or chronic silicosis. Rapid changes in radiographic appearance and cavitation are strong indicators of silicotuberculosis.

Treatment of silicosis is primarily symptomatic - with bronchodilators to relieve airway obstruction, aggressive management of infection with antibiotics and supplemental oxygen as required. Lung transplantation may be considered for patients with end-stage disease. Primary prevention remains the most important intervention; and as a result of better industrial practices, the incidence of silicosis is declining.
